# Exploring the ethics of genetic prioritisation for COVID-19 vaccines

**DOI:** 10.1038/s41431-022-01058-1

**Published:** 2022-03-07

**Authors:** Jago Bruce, Stephanie B. Johnson

**Affiliations:** Wellcome Centre for Ethics and Humanities and Ethox Centre, Nuffield Department of Population Health, University of Oxford, Big Data Institute, Oxford, OX3 7LF USA

**Keywords:** Health policy, Ethics

## Abstract

There is evidence to suggest that host genomic factors may account for disease response variability in COVID-19 infection. In this paper, we consider if and how host genomics should influence decisions about vaccine allocation. Three potential host genetic factors are explored: vulnerability to infection, resistance to infection, and increased infectivity. We argue for the prioritisation of the genetically vulnerable in vaccination schemes, and evaluate the potential for ethical de-prioritisation of individuals with genetic markers for resistance. Lastly, we discuss ethical prioritisation of individuals with genetic markers for increased infectivity (those more likely to spread COVID-19).

## Background

Knowledge as to the role of genetic factors in COVID-19 response is developing [1–8]. There is evidence to suggest that genetic factors may account for disease response variability not already explained by well-established factors such as age, sex, and comorbidities [9]. Enhanced knowledge from, and accessibility of, ‘omic’ technologies mean vaccines are increasingly amenable to individualization or personalization, and the study of immunology and genomics is a growing field [10]. All this means that we may be moving towards an era where genomic information could be combined with other medical and demographic data to produce overall measures of risk [11]. The use of host genomic information is unlikely to be practical, or time- or cost-efficient for implementation during the current COVID-19 pandemic response. However, it is reasonable to assume that as genomic information becomes more readily available to healthcare bodies, and as we move into a more technologically advanced and personalised medical world, genomic information may be of more use in the development of future vaccine allocation schemes where scarce resources must be prioritised. The ethical implications of this have not previously been considered. In this manuscript, we outline some host–genome interactions that have been uncovered for COVID-19, identify some of the ethical concerns they raise, and put forward arguments for how this could be dealt with. We use COVID-19 as an example to explore the topic of genomics and vaccine allocation, but these arguments could be applied to other vaccines.

Specifically, we answer three primary ethical issues that may arise when incorporating genetic risk factors in vaccine allocation policies:

1. Should the genetically vulnerable be prioritised in vaccination allocation schemes?
2. Could there ever be a case for the de-prioritisation of genetically resistant individuals in these schemes?
3. How should genetic information about an individual’s infectivity (likelihood of transmitting to others) be used to inform prioritisation, if at all?

## The role of host genetics in COVID-19 infections

COVID-19 affects individuals differently across populations. Genetic factors may account for disease response variability not already explained by well-established factors such as age, sex, and comorbidities [9]. These factors can affect either the infection dynamics of the individual (the person may be more susceptible to infection or more infectious to others once infected) or the severity of the individual’s infection. Current results suggest that ACE2 and TMPRSS2 gene polymorphisms are both factors that confer significant vulnerability to severe infection [7]. Genome-wide association studies have revealed a number of other loci that may be involved in determining COVID-19 infection severity, including polymorphisms at a particular set of loci on chromosome 3 which have been found to increase the chances of respiratory failure with an odds ratio of 1.77 (ref. [12]). The specific effector gene in this region has recently been narrowed down to *LZTFL1*, a gene with uneven distribution amongst different geographical populations, and it has been suggested that this locus could be investigated as a therapeutic target [8]. Furthermore, there is growing evidence that rare X chromosomally-located variants of TLR7 are associated with severe COVID-19 response in young men [13, 14], and potentially constitute clinically relevant screening targets [15].

Genetic roles in host infectivity (the chance of the individual spreading the infection to others) are less clear. Any factor which leads to increased viral load or severity of infection to the point of coughing and other mechanisms of transmission should affect the host’s chances of transmitting the virus to others [16]. The ‘super-spreader’ phenomenon, wherein certain individuals are unusually likely to transmit disease is recognised as an important factor in disease transmission. The literature on super-spreading has so far focussed on the environmental factors involved, with little attention to genetic factors that may influence whether an individual becomes a ‘super-spreader’ [16, 17]. Despite this, work outside of the field of COVID-19 suggests that genetic factors may play a role in infectivity [18].

As research continues, the role of genetic factors in host response to COVID-19 infection will be further elucidated, but at the time of writing there is sufficient evidence to suggest that genetic factors confer significant risks.

## Ethical principles of vaccine allocation

Several approaches to ethical vaccine distribution have already been outlined in the literature [19–21]. We discuss these below.

## Equality

The World Health Organisation stresses the importance of equality in vaccine prioritisation schemes [22]. It states that: ‘each person’s interest should count equally unless there are good reasons that justify the differential prioritization of resources’; and that within each group in the hierarchy: ‘allocation should aim to promote equality—that is, first come, first served, or random allocation, when no relevant factors distinguish individuals within a particular scheme of allocation’ [22]. In this guidance the importance of promoting equality is acknowledged, but so is the need for a consideration of other principles of allocation in a just prioritisation scheme (an approach based purely on equality would distribute all vaccines based on random allocation, such as by lottery, regardless of the differing levels of need of different individuals). In other words, if we seek to develop a vaccine distribution policy with differential priority given to different individuals or groups then we must appeal to ethical principles other than equality.

## Maximising utility

Utilitarian principles of maximising utility centre on the idea of allocating resources in order to minimise harm or maximise benefits. Utility may be evaluated in differing ways. For example, as the most lives saved or as the most ‘quality-adjusted life years’ gained. In many cases, it is clear that the most good will come from allocating treatment to the worst-off—the most ill or those that suffer the most from a disease are likely to benefit the most from treatment. Alternatively, it could be argued that saving those most likely to survive is a greater use of resources and therefore most likely to bring about the maximum benefit.

## Prioritising the worst-off

The principle of ‘prioritise the worst-off’, as described by Derek Parfit [23, 24], gives more value to benefits concerning worse-off individuals than equal, or larger, benefits to those that are better off. An example of this could be prioritising the elderly and the immunocompromised, who are more likely to suffer from severe COVID-19 but may not respond with maximum efficacy to the vaccine. While this does not appear to be the case for current COVID-19 vaccines, it could apply to future vaccines. Prioritarian arguments could give special consideration to these groups even if the efficacy of the vaccine was very low. ‘Worse-off’ may refer to those with explicit medical vulnerability, such as the immunocompromised, or any other group with increased vulnerability, including those with social risk factors, such as high exposure jobs or crowded housing.

## Reciprocity

Vaccines may be allocated on the basis of reciprocity—the prioritisation of those who society ‘owes’ on the basis of their hard work or other praise-worthy attributes. These arguments are often made in relation to healthcare workers and other frontline workers, as these groups have been subjected to especially high risks and burdens associated with an outbreak or pandemic. The World Health Organisation states that reciprocity (or the ‘principle of prioritising those tasked with helping others’) promotes: ‘the allocation of resources to those who have certain skills or talents that can save many other people, or because something is owed to them on account of their participation in helping others’ [22].

## Main

### 1 - Prioritising the genetically vulnerable

We argue that in principle the genetically vulnerable should be prioritised for a COVID-19 vaccination on the basis that the just allocation of vaccines should prioritise the worst-off and maximise the benefits of vaccination to the population as a whole. In practice this requires vaccinating those most at risk of severe disease and in some circumstances vaccinating others to protect those at most at risk of severe disease.

#### Prioritising those at risk of severe disease

Maximising benefits from vaccine allocation requires that the process benefits those who are more susceptible to severe disease. Prioritising those who are most susceptible to severe disease also prioritises the ‘worse-off’. If vulnerability is defined in terms of increased risk of developing severe COVID-19 for any reason (whether that be medical, social, or demographical) then vulnerability can be both quantified and compared. Thus, where genetic risk is comparable to other clinical and environmental risks it follows that these groups should be treated equally. The statistics support similar prioritisation for the genetically and clinically vulnerable. For example, the odds ratio for severe infection of some chromosome 3 polymorphisms is as high as 1.77 (ref. [7]). The odds ratio of obesity on severe or fatal COVID-19 infection is currently thought to be similar, at 1.72 (ref. [25]). So long as our aim is to prioritise the health of the vulnerable, and vulnerability is defined as statistical risk of severe COVID-19 infection, then discrimination between different types of risk factors is not justified. As such, genetic vulnerability should be prioritised in vaccination schemes alongside other kinds of vulnerability according to the statistical risk conveyed.

#### Protecting the vulnerable by vaccinating others

Protecting the most vulnerable from severe COVID-19 infection may not always mean giving them the vaccine first. Policies that deviate from allocating vaccines to the most clinically vulnerable can be justified under both prioritarian and utilitarian views, so long as they are successful in protecting at-risk groups. Notably, vaccinating the vulnerable is only an effective strategy for protecting the vulnerable if the vaccine remains effective in these groups. This is not always the case. Flu vaccines, for example, are much less effective in the elderly than they are in younger population groups [26, 27]. Williams et al. [28] refer to vaccinating one group to protect another, as ‘indirect protection strategies’. Such strategies extend protection beyond the individual receiving the vaccination (though the vaccinated individual may benefit from it), because of reduced transmission of disease more generally and to at-risk groups in particular [28].

Current models have suggested that an ‘oldest-first’ vaccination strategy (which is closest to our hypothetical strategy of vaccinating the most vulnerable first) is more effective than a ‘youngest-first’ strategy for COVID-19 (ref. [29]). Even so, there is a possibility that in future pandemics, protecting the vulnerable will not be best achieved through immediate vaccination of these groups, but instead through the vaccination of less at-risk groups such as the young to reduce disease prevalence and spread, and to increase population immunity. This would be dependent on vaccination reducing transmission in the population. This has three implications for the use of genomic information in vaccine prioritisation: (1) if a COVID-19 vaccine is not efficacious in the genetically vulnerable to severe infection, then it is possible that they should not be prioritised; (2) vaccinating others first to protect the genetically vulnerable should be considered; (3) vaccinating those with higher infectivity due to genetics may be justified (discussed further below).

### 2 - De-prioritisation of the genetically resistant

De-prioritisation may appear to be ethically equivalent to prioritisation. In any case of prioritisation, when an individual is prioritised, those that this individual has passed when moving ‘up the list’ can be thought to be moving ‘down’ in response. Conversely, the act of de-prioritising brings many individuals ‘up’ the priority list. Given this, it could be argued that such priority-shifting movements are ethically equivalent whether individuals are moved ‘up’ or ‘down’ the list, so long as both are justified on reasonable grounds (such as maximising benefits or prioritising the most vulnerable) as they both result in similar net changes across the population as a whole. Below, we consider three specific groups where genetic de-prioritisation could be used in practice: marginalised ethnic groups; healthcare workers; and already low-risk individuals. We argue that de-prioritisation of genetically resistant individuals, while justifiable in some cases, is unlikely to yield sufficient benefits to make it desirable in most cases and may be ethically problematic in others.

#### De-prioritisation of marginalised groups of shared genetic ancestry

De-prioritisation has potential for unjustified discrimination against certain groups. Significant resistance markers could be exclusive to, and highly prevalent within, groups of a shared genetic ancestry. For example, the gene cluster conferring vulnerability to severe COVID-19 infection located at the locus on chromosome 3 has been found to be distributed unevenly across geographical population groups [30]. In fact, over 60% of individuals with South-Asian ancestry are thought to have the risk-carrying variants, compared to just 15% in European ancestry groups [8]. It is unknown what other variations might be found, and in which populations, going forward.

In their recent paper, Walker et al. consider the use of genetic information in the context of Hepatitis C Virus treatment prioritization [31]. They argue, and we agree, that de-prioritisation of already marginalised groups is a social injustice as it disadvantages individuals that are already at a disadvantage within the population. Any vaccination scheme that de-prioritised an already socially-disadvantaged group on the basis of genetic information would, therefore, be unjust.

#### De-prioritisation of healthcare workers and first responders

Healthcare workers (HCWs) and first responders are likely to consistently be among the very first to receive the COVID-19 vaccine. They are often prioritised for three reasons. First, under prioritarian principles they are worse-off because of the risks associated with high occupational exposure to the virus. Second, vaccinating HCWs may slow the spread of infection, as they are at high risk of being transmission vectors. Vaccinating HCWs may also confer similar benefits indirectly by minimising their absence from work and reducing strain on essential healthcare services. Third, they are owed the vaccine on the basis of reciprocity.

De-prioritising genetically resistant HCWs may be ethically justified as their reduced susceptibility to disease invalidates all three grounds for their prioritisation. If HCWs are resistant to severe COVID-19 infection they are no longer ‘worse-off’ and no longer at high risk of being unable to work due to COVID-19 infection. They are likely to also have a reduced capacity for transmitting the virus. Lastly, while genetically resistant HCWs may not have encountered significant physical risks in their line of work, it may be true that HCWs have incurred increased burdens such as increased working hours. Vaccination, however, has limited value as a ‘reward’, as they are already at significantly less risk of severe infection. Persad et al. argue that if we are to reward worthy conduct then there are other, perhaps more appropriate, benefits that can be offered to recognise worthy conduct, such as financial benefits [20].

However, an ethical risk to the use of genomic information for vaccine prioritisation in the case of HCWs (and, to a lesser extent, any group) is that genomic testing may bring about workplace discrimination. As mentioned above, Gyngell et al. argue that while clear cases of discrimination based on genotype are illegal in many parts of the world, genetic testing can also lead to more subtle forms of discrimination and coercion [11]. Those identified as being resistant to severe COVID-19 may feel more pressure to undertake high-risk activities and shield other workers from infection, creating additional social expectations which constrain their freedom of choice [11]. Placing restraints on the disclosure and use of genomic information obtained as part of vaccine prioritisation could help to mitigate this risk.

Overall, while there is a case for de-prioritising HCWs with a substantially low risk of severe COVID-19 infection, the extent to which HCWs may be ethically de-prioritised would depend on the nature of the resistance conveyed, and the efficacy of the vaccine in reducing not only severity of illness, but transmission. The benefits of such an act would also have to be balanced with any potential for discrimination.

#### De-prioritisation of already low-risk individuals

Lastly, we consider those of a ‘lowest’ priority level who do not meet other criteria that prioritises them for a vaccine. For those who are found to be genetically resistant within this group, there is unlikely to be much useful benefit in de-prioritisation, particularly as the resistant group is likely to be in the minority. However, if there were significant vaccine supply shortage it may be justifiable to further sub-divide the lowest priority group.

### 3 – Prioritising those with higher infectivity, but low susceptibility to severe disease

Unlike other resources such as ventilators or hospital beds, vaccines are unique in that they benefit not only the recipient but also those around them. Vaccinating those with higher infectivity, but less susceptibility, to severe disease confers benefits to the public’s health with little accompanying benefit for the individual, whilst the risks and side-effects of vaccination remain the same. In the case of influenza, for example, a child may have very little risk of severe illness but could be particularly infective should they contract the virus, the ethics of which have previously been considered [32]. The child stands to gain very little from the vaccine but may still suffer a negative reaction to the vaccination. A similar scenario could arise in COVID-19 with regards to genetic susceptibility to infection, and infectivity. While low susceptibility and high infectivity individuals cannot be prioritised on the basis of their own risk of disease, vaccinating them could lead to reductions in the number of COVID-19 cases and associated deaths. In other words, it could further the protection of the most vulnerable to severe disease from COVID-19 infection. Thus, in the protection of the vulnerable from infection, those that are not themselves particularly vulnerable to COVID-19 but have higher infectivity should be prioritised, although this would take a lower place in the vaccination hierarchy than those who are both highly infective and highly susceptible (worse-off).

The question of mandatory vaccination of these groups is pertinent, although beyond the scope of this paper. Arguments for mandatory vaccination rest on the increased risk of harm to others that accompanies the decision not to get vaccinated [33–35].

## Conclusions

We have outlined the place that genomics could have within COVID-19 vaccine allocation schemes. We argue that those vulnerable to severe disease should be prioritised according to their relative risks, and that genetic risk factors should be treated equivalently to other risk factors in this regard. De-prioritising the genetically resistant can be justified ethically but requires careful balancing of the potential for discrimination against any benefits arising from such a scheme. Lastly, we have argued that the vaccination of individuals that are not at risk themselves but have genetic factors that make them more infective should they contract the virus is justified, on the grounds that this will protect more vulnerable individuals by reducing transmission.
